# *Mycobacterium canettii* Infection of Adipose Tissues

**DOI:** 10.3389/fcimb.2017.00189

**Published:** 2017-05-17

**Authors:** Fériel Bouzid, Fabienne Brégeon, Isabelle Poncin, Pascal Weber, Michel Drancourt, Stéphane Canaan

**Affiliations:** ^1^Aix-Marseille Université, CNRS, EIPL IMM FR3479Marseille, France; ^2^Aix Marseille Université, URMITE, UMR CNRS 7278, IRD 198, INSERM 1095, IHU Méditerranée InfectionMarseille, France; ^3^Service des Explorations Fonctionnelles Respiratoires, Centre Hospitalo-Universitaire Nord, Pôle Cardio-Vasculaire et Thoracique, Assistance Publique Hôpitaux de MarseilleMarseille, France

**Keywords:** tuberculosis, *Mycobacterium canettii*, *Mycobacterium tuberculosis*, adipose tissues, adipocyte

## Abstract

Adipose tissues were shown to host *Mycobacterium tuberculosis* which is persisting inside mature adipocytes. It remains unknown whether this holds true for *Mycobacterium canettii*, a rare representative of the *M. tuberculosis* complex responsible for lymphatic and pulmonary tuberculosis. Here, we infected primary murine white and brown pre-adipocytes and murine 3T3-L1 pre-adipocytes and mature adipocytes with *M. canettii* and *M. tuberculosis* as a positive control. Both mycobacteria were able to infect 18–22% of challenged primary murine pre-adipocytes; and to replicate within these cells during a 7-day experiment with the intracellular inoculums being significantly higher in brown than in white pre-adipocytes for *M. canettii* (*p* = 0.02) and *M. tuberculosis* (*p* = 0.03). Further *in-vitro* infection of 3T3-L1 mature adipocytes yielded 9% of infected cells by *M. canettii* and 17% of infected cells by *M. tuberculosis* (*p* = 0.001). Interestingly, *M. canettii* replicated and accumulated intra-cytosolic lipid inclusions within mature adipocytes over a 12-day experiment; while *M. tuberculosis* stopped replicating at day 3 post-infection. These results indicate that brown pre-adipocytes could be one of the potential targets for *M. tuberculosis* complex mycobacteria; and illustrate differential outcome of *M. tuberculosis* complex mycobacteria into adipose tissues. While white adipose tissue is an unlikely sanctuary for *M. canettii*, it is still an open question whether *M. canettii* and *M. tuberculosis* could persist in brown adipose tissues.

## Introduction

In mammals, brown adipose tissue (BAT) and white adipose tissue (WAT) are mainly composed of mature adipocytes, pre-adipocytes and immune cells (Ouchi et al., 2011). WAT and BAT differ in cell morphology, tissue distribution and physiological functions (Gomez-Hernandez et al., 2016): WAT constitutes the main energy reserve of the organism while BAT ensures thermogenesis in hibernating animals and newborns (Cannon and Nedergaard, 2004; Gomez-Hernandez et al., 2016) and remains metabolically active in adults (Nedergaard et al., 2007).

Adipose tissues have been previously supposed to act as long-term sanctuaries sheltering *Mycobacterium tuberculosis*, the major agent of human tuberculosis worldwide (Neyrolles et al., 2006; Kim et al., 2011; Agarwal et al., 2014, 2016; Rastogi et al., 2016). *In vitro* experiments were conducted using murine 3T3-L1 mature adipocytes (Neyrolles et al., 2006; Kim et al., 2011; Rastogi et al., 2016), and 3T3-L1 pre-adipocytes (Neyrolles et al., 2006), but also with murine primary pre-adipocytes and mature adipocytes obtained from WAT (Agarwal et al., 2014). In immune-competent mice, intravenously and intra-nasally inoculated *M. tuberculosis* disseminates in WAT of visceral, subcutaneous, peri-renal and mesenteric adipose depots (Agarwal et al., 2014, 2016). In these models, the burden of *M. tuberculosis* in WAT depots stagnated or decreased with time (Agarwal et al., 2014). Indeed, compelling data show that *M. tuberculosis* adopts in mature adipocytes and WAT a non-replicating, dormant state characterized by an arrest of multiplication, accumulation of intra-cytosolic lipid inclusions (ILIs), resistance to anti-mycobacterial drugs (Neyrolles et al., 2006; Agarwal et al., 2014) and up-regulation of *dosR* and *icl* genes (Rastogi et al., 2016). The infection of mature adipocytes was therefore proposed as a suitable model to study the accumulation of neutral lipids into ILIs within mycobacteria (Santucci et al., 2016).

Among the *M. tuberculosis* complex (MTBC), *Mycobacterium canettii* is a peculiar member specifically diagnosed in dozens of tuberculosis patients with reported contacts to the Horn of Africa (Aboubaker Osman et al., 2015). *M. canettii* differs from the other members of the MTBC by processing a larger 4.48 ± 0.05 Mb mosaic genome and producing cordless and smooth-looking mycobacteria (Gutierrez et al., 2005; Koeck et al., 2011; Supply et al., 2013; Boritsch et al., 2016a) with an ability of intra-species horizontal gene transfer (Boritsch et al., 2016b). *M. canettii* infection mainly presents as lymph node and pulmonary tuberculosis (Koeck et al., 2011; Aboubaker Osman et al., 2015). However, *M. canettii* pulmonary tuberculosis is unique in being seemingly non-contagious (Koeck et al., 2011).

In order to further examine the *in-vitro* interactions of *M. canettii* with natural adipose tissues, we developed *in-vitro* experimental models using primary murine white and brown pre-adipocytes; and further investigated the interactions between *M. canettii* and murine 3T3-L1 pre-adipocytes and mature adipocytes using *M. tuberculosis* H37Rv as a positive control.

## Materials and methods

### Mycobacteria

*M. tuberculosis* H37Rv and *M. canettii* CIPT 140010059 were used in this study. Mycobacteria were cultivated in Middelbrook 7H10 (Becton Dickinson, Le Pont de Claix, France) supplemented with 10% oleic acid-albumin-dextrose-catalase (OADC) (Becton Dickinson). Fluorescent *M. canettii* CIPT 140010059 mCherry (CSURP3621) was constructed by transforming the pMV261 mCherry vector (gift from L. Kremer, CPBS, Montpellier, France) into the *M. canettii* strain (Alibaud et al., 2011). Fluorescent strains were cultured in Middlebrook 7H10 broth supplemented with 10% OADC and 50 μg/mL kanamycin (Sigma). Prior to infection, mycobacteria were resuspended in phosphate buffered saline (PBS), shaken on a vortex mixer for 10 min with glass ball to disperse clumps and centrifuged for 1 min at 300 g to remove residual clumps. The supernatant was then dispersed by expelling the suspension 10 times through a sterile 25-gauge needle attached to a 1-mL syringe. Calibration was then performed according to Mcfarland standard confirmed by counting mycobacteria after Ziehl-Neelsen staining (RAL diagnostics, Martillac, France). All experiments using these mycobacteria were performed in a biosafety level 3 laboratory of the Faculté de Médecine, Aix-Marseille Université, France.

### Primary murine pre-adipocyte culture

The experiments conducted on mice was approved by the Institutional Animal Care and Use Committee of Aix-Marseille University “C2EA-14,” France and registered by the “Ministère de l'Enseignement Supérieur et de la Recherche” under reference n° 2015092415474605. Mice were handled according to the rules of Décret N° 2013–118, Février 7, 2013, France. Inguinal WAT and inter-scapular BAT were collected from ten 6-week-old Balb/cByj mice. Pre-adipocytes cells were isolated from tissues as described elsewhere (Aune et al., 2013) and then cultured in complete culture medium Dulbecco's Modified Eagle Medium DMEM/F12 (Invitrogen, France) supplemented with 10% heat-inactivated fetal bovine serum (FBS) and were plated at a concentration of 5 × 10^4^ cells/ml/well in 12-well plates and incubated at 37°C under a 5% CO_2_ atmosphere.

### 3T3-L1 cell lines

Murine embryonic fibroblasts 3T3-L1 (ATCC, lot 62158491) were cultured in DMEM (Invitrogen, France) supplemented with 10% heat-inactivated FBS at 37°C under a 5% CO_2_ atmosphere. Prior to confluence, cells were plated at 5 × 10^4^ cells/well in a 12-well sterile plates and incubated as described above. Six days after plating, pre-adipocytes reach confluency and stop dividing. Adipocyte differentiation was initiated upon confluency by adding 1 μg/mL insulin (Sigma), 0.5 mM isobuthyl-methylxanthine (Sigma), and 1 μM dexamethasone (sigma) into the culture medium. After 48 h of induction, the medium was replaced by DMEM-10% FBS containing 1 μg/mL of insulin and maintained each 2 days in order to achieve a full differentiation as previously described (Neyrolles et al., 2006). Adipocyte differentiation was checked by BODIPY 493/503 staining (Sigma) of lipid droplets over the 2-week experiments.

### Infection

Pre-adipocytes and mature adipocytes were infected with a multiplicity of infection (MOI) of 1 per cell at day 6 after plating and at day 10 post-induction respectively. Accordingly, 1 × 10^5^ cells per well were infected with 1 × 10^5^ mycobacteria. After 4-h incubation, cells were washed 3 times with serum-free DMEM to remove all extracellular bacteria. Only mature adipocytes were then incubated with 200 μg/mL amikacin for 2 h and then washed thrice. The last wash was plated to validate the correct elimination of extracellular mycobacteria. The inoculated cultures were incubated with fresh complete medium up to 7 days for primary cultures and 12 days for 3T3-L1 cell lines at 37°C under a 5% CO_2_ atmosphere. At various time points, infected cells were lysed in PBS containing 0.1% Triton X-100 and the lysate was plated at 10-fold dilutions from 10^−1^ to 10^−4^ onto Middlebrook 7H10 agar plates incubated at 37°C under a 5% CO_2_ atmosphere. The number of colonies enumerated after 20-day of incubation was used to estimate the number of colony-forming units (CFUs) per 10^5^ cells. All experiments were conducted in triplicate: three independent plates the same day for primary cell infection experiments and three replicates at different days with different bacterial cultures for 3T3-L1 cells infection experiments.

### Intracellular localization of mycobacteria

Ziehl-Neelsen staining (RAL diagnostics, Martillac, France) was performed on infected cells (MOI 1:1 and 5:1) at day 3 p.i. Observations were acquired using the slide scanner Axio Scan.Z1 (Zeiss) (Magnification 20X) with a color Hitachi tri-CCD (1,800 × 1,200 pixels) camera. The number of intracellular mycobacteria per one cell was recorded on image acquisition by ImageJ software.

*M. canettii* mCherry was used to localize intracellular bacteria using confocal microscopy. Infected adipocytes (MOI 10) were fixed using 4% formaldehyde at day 3 p.i. Lipid droplets were stained with BODIPY 493/503 (Sigma). The images were acquired using a confocal microscope (Zeiss, Spinning disk with a Yokogawa head and an emCCD camera 512 × 512 pixels) at magnification 63X/1.4 oil objective.

### Processing for electron microscopy

Cells were fixed at room temperature with 2.5% glutaraldehyde in Na-cacodylate buffer 0.1 M (pH 7.2) containing 0.1 M sucrose, 5 mM CaCl_2_, and MgCl_2_ 5 mM, washed with complete cacodylate buffer and postfixed for 1 h at room temperature with 1% osmium tetroxide in the same buffer without sucrose (de Chastellier, 2008). They were washed with buffer, scratched gently, concentrated in agar up to 2% with cacodylate buffer and processed for 1 h at room temperature with 1% uranyl acetate in maleate buffer. The samples were dehydrated in a graded series of ethanol solutions and gradually incorporated in Spurr resin. Thin sections (80 nm thick) were stained with 1% uranyl acetate in distilled water and then with lead citrate before being observed by electron microscopy.

### Nile red staining

Adipocytes were infected with *M. canettii* at MOI 5:1 on 12-well plate. At day 7 p.i. all wells were lysed in 500 μL water containing 0.1% Triton X-100. The pooled lysate was sonicated and centrifuged at 9,000 g for 25 min. The *M. canettii* cells were washed thrice with 0.1% Triton X-100 and the pellet was resuspended in 500 μL of PBS containing 0.05% Tween 80. Nile red staining was performed as previously described (Christensen et al., 1999). The stained smears were subjected to high resolution confocal (Zeiss AiryScan head, magnification 63X, Numerical aperture = 1.4), with higher resolution (1.6X) than conventional confocal.

### Lactate dehydrogenase (LDH) assay

LDH, a soluble cytoplasmic enzyme released into extracellular space when the cell membrane is damaged, was used as an estimator of cell lysis (Korzeniewski and Callewaert, 1983). Culture supernatants of uninfected or infected mature adipocytes with *M. canettii* or *M. tuberculosis* (MOI 1) were collected at day 3, 7, and 12 post-inoculation. The release of LDH from negative controls and infected cells was measured spectrophotometrically at 340 nm on a Cobas 8000 (Roche, Meylan, France).

### Statistical analyses

Statistics were performed using the SigmaPlot13 software. The distribution of the variables was assessed statistically with the Shapiro-Wilk test. The equality of the variance was checked using the Brown-Forsythe test. All data were normally distributed and were expressed using means ± standard deviation. The statistical significance was performed using the Student *T*-test. The Chi-square test was used to compare rates and proportions with Yates correction. A *p* <0.05 was regarded as significant.

## Results

### *M. canettii* and *M. tuberculosis* within primary murine pre-adipocytes

**Figure 1:**
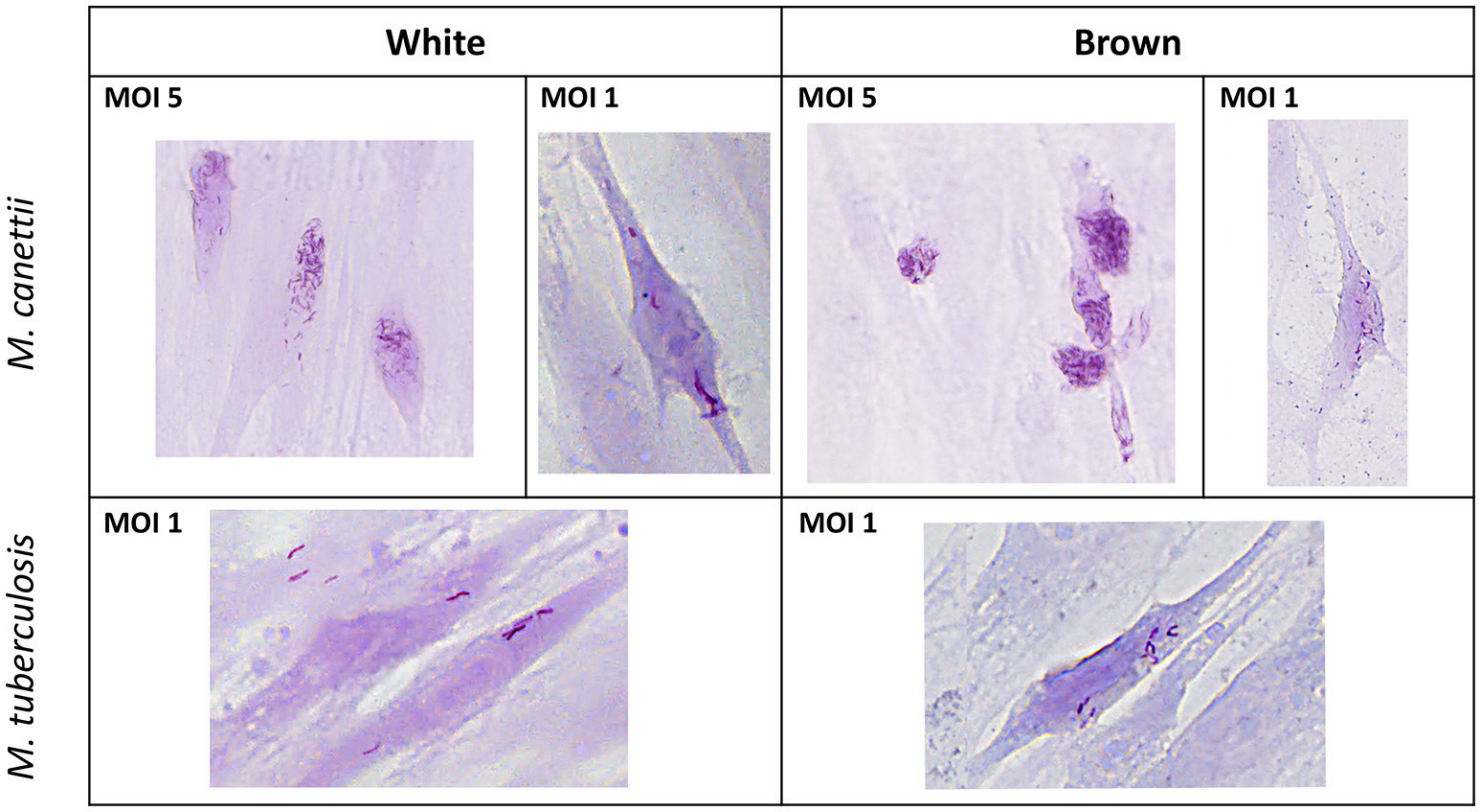
**Ziehl-Neelsen staining performed on infected primary murine pre-adipocytes**. White and brown pre-adipocytes obtained from *ex vivo* culture from mouse adipose depots were infected for 4 h with *M. canettii* CIPT 140010059 or *M. tuberculosis* H37Rv and stained at day 3 post-inoculation. Microscopic observations at 20x magnification showed infection of white and brown pre-adipocytes by *M. canettii* upper panel and by *M. tuberculosis* H37Rv lower panel.

Pre-adipocytes purified from murine inguinal WAT and inter-scapular BAT were *ex vivo* inoculated with *M. canettii* or *M. tuberculosis* for 4 h p.i. For all experiments, extracellular mycobacteria were removed by extensive washing with serum-free medium, 4 h p.i. At day 3 p.i. Ziehl-Neelsen staining was performed to assess the ability of mycobacteria to infect primary murine pre-adipocytes. While negative control cells remained free of detectable mycobacteria, microscopic observations clearly showed intracellular *M. canettii* and *M. tuberculosis* in white and brown pre-adipocytes (Figure 1).

The ratio of infected cells relative to the total number of observed cells as well as the average number of intracellular bacilli per one cell were measured on a random selection of 10 microscope fields on stained infected cells at day 3 p.i. (see Supplementary Data S1). White pre-adipocytes infected at MOI 1:1 yielded 19% and 18% of infected cells with 9 ± 6 *M. canettii* bacilli per cell and 7 ± 5 *M. tuberculosis* organisms per cell, respectively. Likewise, 22 and 19% of brown pre-adipocytes were infected with 16 ± 7 *M. canettii* organisms/cell and 11 ± 4 *M. tuberculosis* organisms/cell, respectively. The ratio of infection did not significantly differ between brown and white pre-adipocytes (*p* = 0.7 for *M. canettii* and *p* = 0.8 for *M. tuberculosis*, χ^2^ test) but the number of bacilli in brown pre-adipocytes was significantly higher than in white pre-adipocytes (*p* = 0.02 for *M. canettii* and *p* = 0.03 for *M. tuberculosis*).

**Figure 2:**
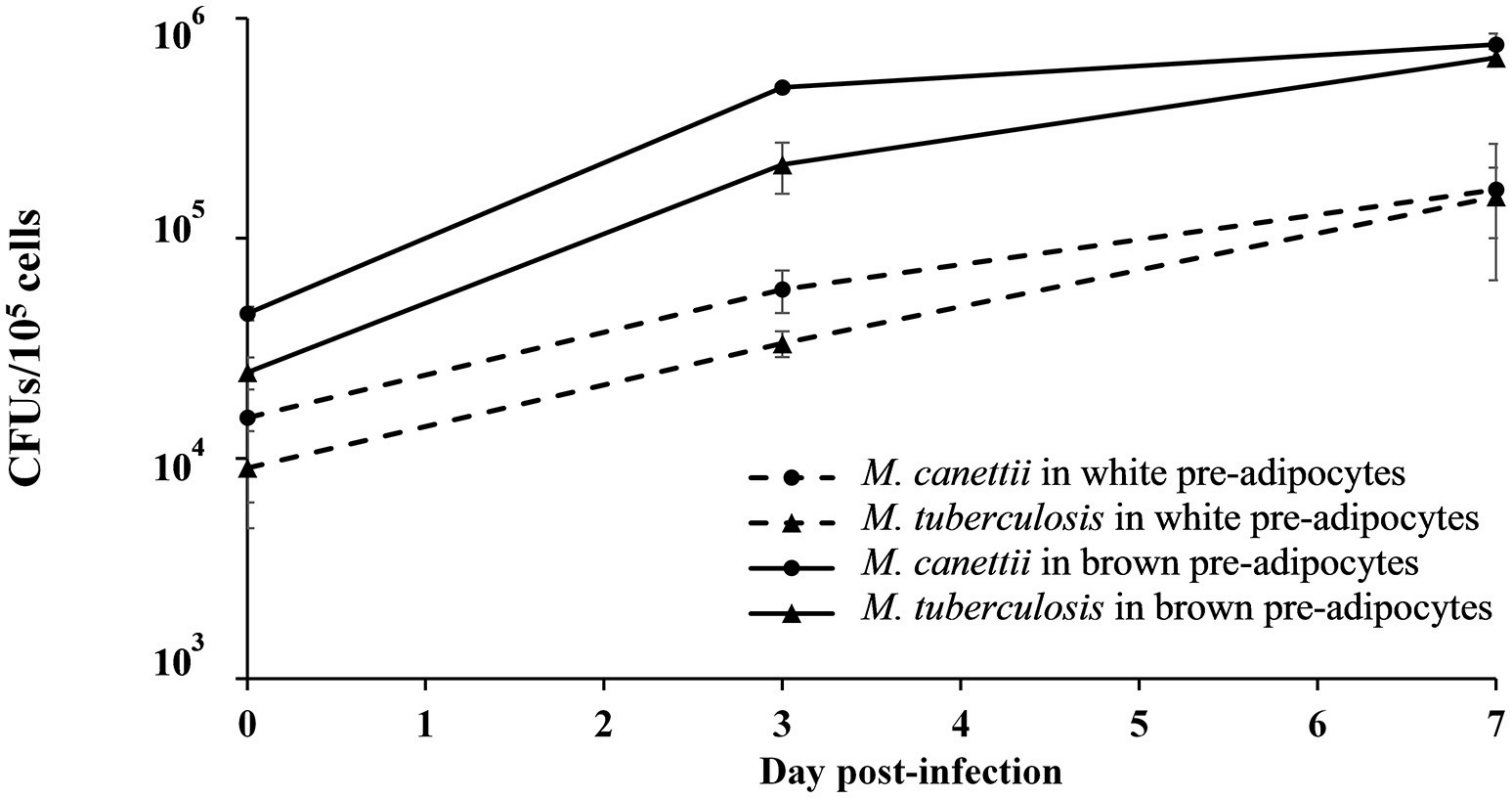
**Number of colony-forming units (CFUs/10^**5**^ cells) of ***M. canettii*** and ***M. tuberculosis*** in murine white and brown pre-adipocytes over the 7-day experiments**. Primary murine pre-adipocytes were infected at MOI 1:1 with *M. canettii* CIPT 140010059 or *M. tuberculosis* H37Rv for 4 h. At day 0, 3, and 7 post-inoculation, CFUs were scored by plating serial dilutions of cell lysates after washing three times to remove of extracellular mycobacteria. Results shown are the mean of three independent experiments and bars indicate + standard deviation. A similar intracellular kinetic was observed for the two mycobacteria in both type cells.

In order to confirm these observations and to study the viability of bacilli into the infected cells, the number of intracellular mycobacteria was quantified by scoring CFUs/10^5^ cells after plating infected cell lysates with *M. canettii* or *M. tuberculosis* at MOI 1:1 at different time intervals (Figure 2) (see Supplementary Data S1). At day 0, white pre-adipocytes had internalized 15% (1.5 × 10^4^ ± 9 × 10^3^ CFUs) and 10% (9 × 10^3^ ± 4 × 10^3^ CFUs) of the initial 1 × 10^5^ inoculum of *M. canettii* and *M. tuberculosis*, respectively. As for brown pre-adipocytes, 45% (4.5 × 10^4^ ± 2.3 × 10^3^ CFUs) of the *M. canettii* inoculum and 25% (2.47 × 10^4^ ± 4 × 10^3^ CFUs) of the *M. tuberculosis* inoculum was phagocytized. By scoring CFUs, intracellular *M. canettii* and *M. tuberculosis* organisms were more abundant in primary brown pre-adipocytes than in white pre-adipocytes (*p* = 0.01 for *M. canettii* and *p* = 0.008 for *M. tuberculosis*) which is consistent with our microscopic observations.

A similar intracellular behavior was observed for *M. canettii* and *M. tuberculosis* during the 7-day experiment in primary white and brown pre-adipocytes: both mycobacteria survived and multiplied with approximately one log gain.

### Interactions between *M. canettii* and 3T3-L1 pre-adipocytes and mature adipocytes

#### *M. canettii* is internalized into 3T3-L1 pre-adipocytes and mature adipocytes

To explore *M. canettii* interactions with 3T3-L1 pre-adipocytes and mature adipocytes compare to *M. tuberculosis* (Neyrolles et al., 2006; Kim et al., 2011), the quantification of the ratio of infected cells/total cells and the number of intracellular bacilli/cell were performed on a random selection of 10 microscope fields after Ziehl-Neelsen staining of infected cells at day 3 p.i. (see Supplementary Data S1). For pre-adipocytes, 20% of cells were infected with 7 ± 5 *M. canettii* organisms/cell while 40% of cells were infected with 12 ± 6 *M. tuberculosis* organisms/cell which is significantly higher compared to *M. canettii* infection (*p* <0.001, χ^2^ test). Under the same conditions, only 9% and 17% of adipocytes were infected with *M. canettii* and *M. tuberculosis*, respectively. The ratio of infected cells was significantly higher for pre-adipocytes compared to mature adipocytes (*p* = 0.002 for *M. canettii* and *p* <0.001 for *M. tuberculosis*, χ^2^ test).

**Figure 3:**
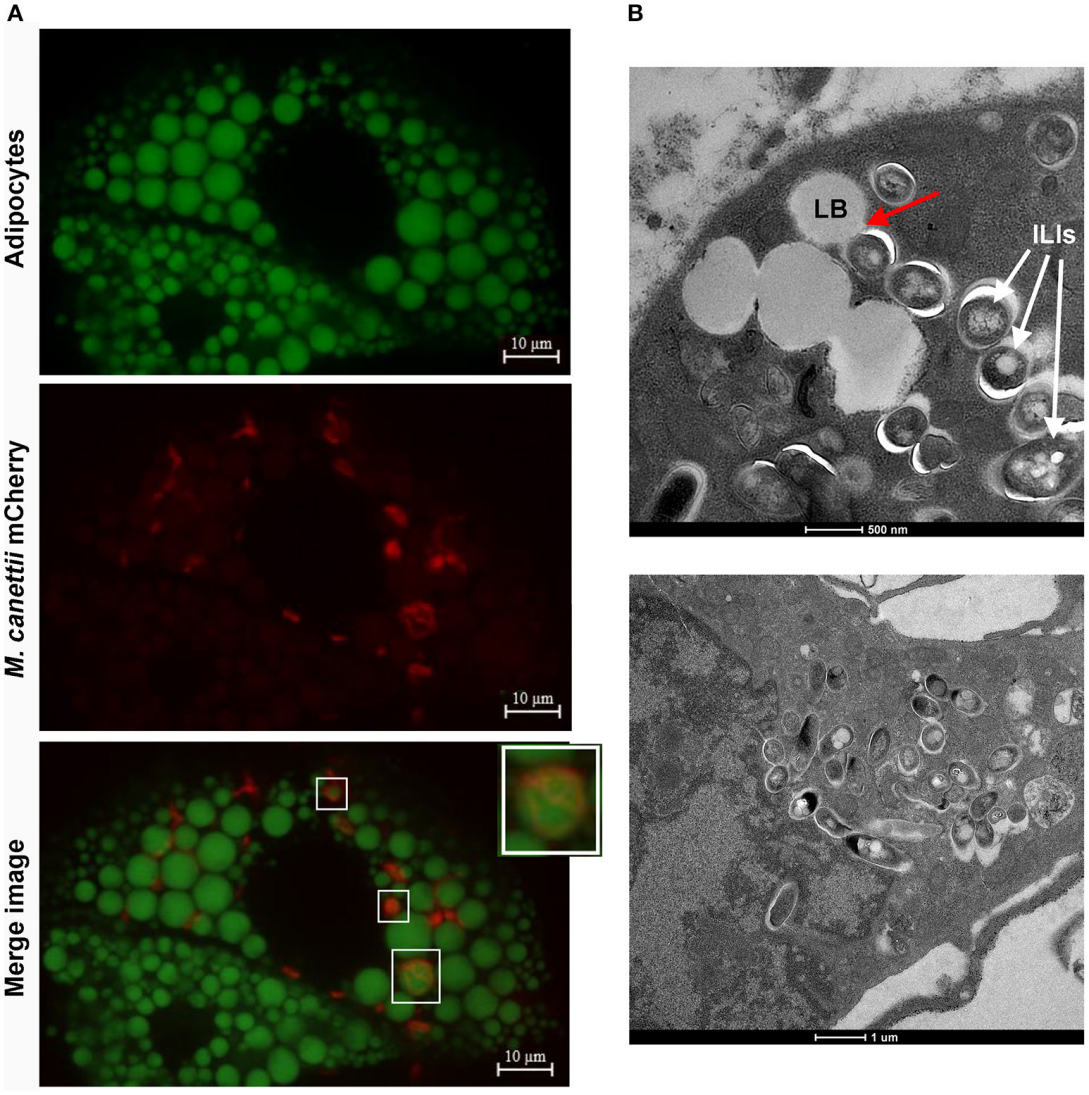
*****M. canettii*** infection of 3T3-L1 mature adipocytes. (A)** Confocal images of 3T3-L1 mature adipocytes infected with *M. canettii*. Adipocytes were infected at MOI 10:1 with *M. canettii* mCherry for 4 h. At day 3 post-inoculation, extracellular mycobacteria were washed off, infected cells were fixed in 4% formaldehyde and the lipid droplets of adipocytes were stained using BODIPY 493/503. Confocal images showed intracellular location of *M. canettii* mCherry and 30% of intracellular bacilli were next to lipid bodies (White squares). **(B)** Electron microscopy observations of 3T3-L1 mature adipocytes infected with *M. canettii*. Adipocytes were infected at MOI 5:1 with *M. canettii* for 4 h. At day 3 post-inoculation, the supernatant was removed and infected cells were fixed for electron microscopy. *M. canettii* organisms were inside adipocytes in close contact with adipocytes LBs as indicated by the red arrow and accumulated ILIs (upper panel). Pre-adipocytes that failed to reach full differentiation were infected also with *M. canettii* (lower panel).

To confirm the intracellular location of *M. canettii* within 3T3-L1 mature adipocytes, cells were inoculated with *M. canettii* CIPT 140010059 mCherry (CSURP3621) at MOI = 10. Three days p.i. infected cells were treated with BODIPY 493/503 stain and fixed. Confocal microscopy observations showed the presence of 15 ± 6 intracellular *M. canettii* inside adipocytes, as compared to negative controls which remained free of detectable mCherry-fluorescence (Figure 3A). We noticed that approximately 30% of intracellular *M. canettii* organisms were localized in close contacts with cytoplasmic lipid bodies (LBs) in 100% of the observed adipocytes (Figure 3A white squares).

Further electron microscopy observation of adipocytes infected at a MOI 5:1 for 3 days demonstrated the presence of *M. canettii* within adipocytes in close contact with adipocyte LBs, corroborating observations performed by confocal microscopy (Figure 3B, upper panel). Intracellular *M. canettii* bacilli were loaded with small electron-dense vesicles resembling ILIs (Figure 3B, upper panel). We also found that some pre-adipocytes that failed to reach full differentiation were infected by *M. canettii* which had weakly accumulated ILIs (Figure 3B, lower panel).

#### Intracellular outcome of internalized *M. canettii*

**Figure 4:**
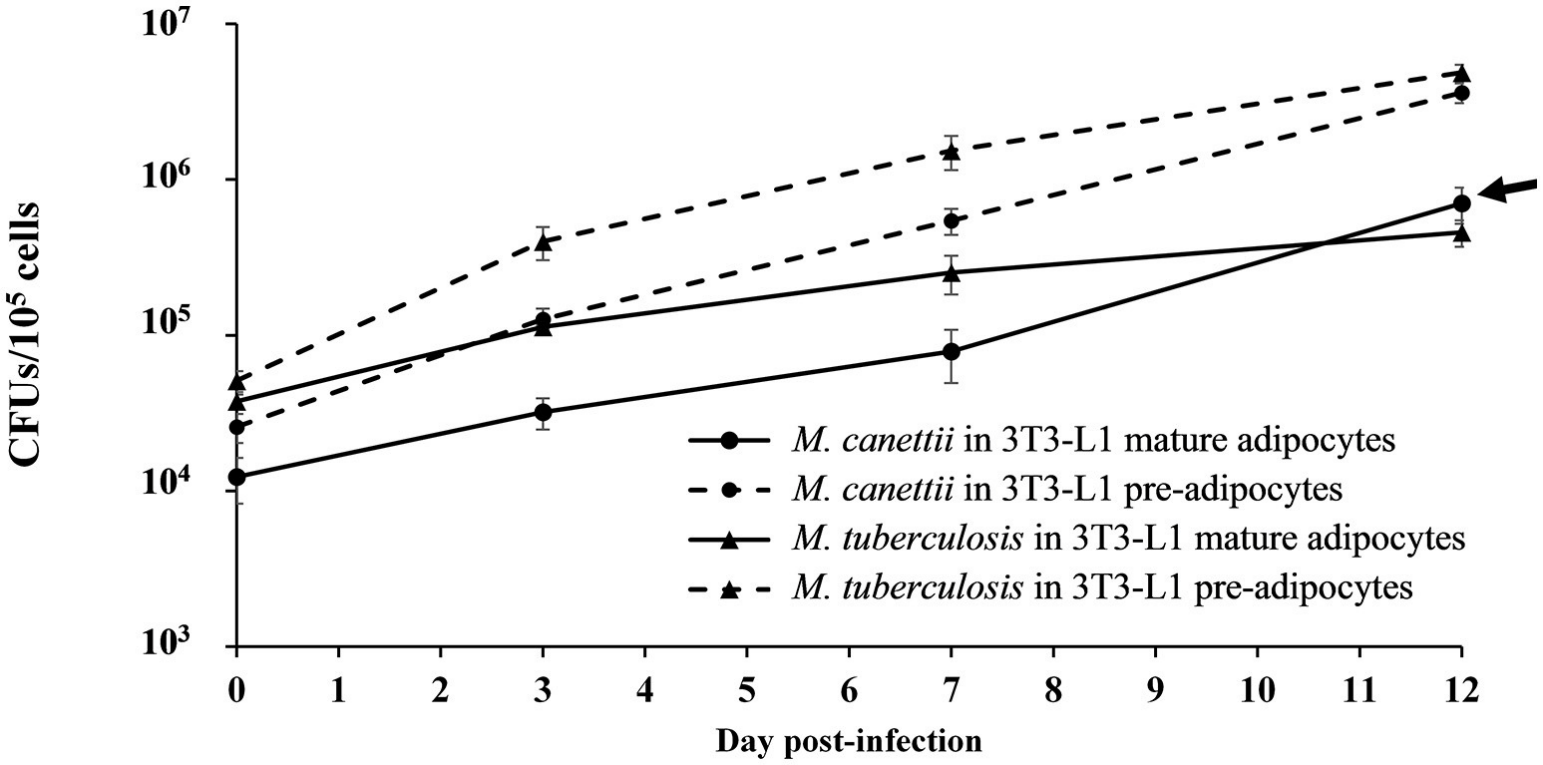
**Intracellular kinetic of ***M. canettii*** CIPT 140010059 and ***M. tuberculosis*** H37Rv in 3T3-L1 pre-adipocytes and adipocytes during 12-day experiments**. Cells were infected at MOI 1:1 with *M. canettii* or *M. tuberculosis* for 4 h. The number of bacterial colony forming units (CFU) was determined at the indicated time points (0, 3, 7, and 12 day p.i.). A similar intracellular kinetic was observed for *M. canettii* and *M. tuberculosis* inside pre-adipocytes. However, in mature adipocytes *M. tuberculosis* stopped its replication between day 3 and 12 day p.i. while *M. canettii* continued to replicate (arrow). Data shown are the mean ± standard deviation of three independent experiments.

Using the 3T3-L1 continuous cell line which is more robust and easier to manipulate than primary cells, it was possible to compare the successive steps of infection over 12 days in three independent experiments conducted at different days, comparing pre-adipocytes and mature adipocytes. After thorough dispersal of the mycobacterial aggregate, cells were infected with *M. tuberculosis*, used as positive control, or *M. canettii* at a MOI 1:1 for 4 h followed by extensive elimination of extracellular mycobacteria. All results were compared with non-infected cells manipulated in parallel, which remained negative in culture during the 12-day experiment. At various time points 0, 3, 7, and 12 days p.i. cells were lysed and viable bacterial content was measured by plating cell lysates onto Middelbrook agar 7H10 and scoring CFUs/10^5^ cells (Figure 4) (see Supplementary Data S1).

As for internalization, at day 0, pre-adipocytes contained 25% of the initial *M. canettii* inoculum (2.5 × 10^4^ ± 5 × 10^3^ CFUs) and 50% of the initial *M. tuberculosis* inoculum (5 × 10^4^ ± 8 × 10^3^ CFUs). In mature adipocyte infection experiments, 12% (1.2 × 10^4^ ± 4 × 10^3^ CFUs) of the infecting *M. canettii* inoculum and 37% of the infecting *M. tuberculosis* inoculum (3.7 × 10^4^ ± 4 × 10^3^ CFUs) was internalized. Initial infection of mature adipocytes was significantly lower with *M. canettii* (*p* = 0.001) than with *M. tuberculosis*, as observed with infected pre-adipocytes (*p* = 0.006), but numbers of intracellular mycobacteria from day 0 were significantly higher in pre-adipocytes than in mature adipocytes (*p* = 0.01 for *M. canettii* and *p* = 0.03 for *M. tuberculosis*).

As for intracellular behavior, both mycobacteria continuously replicated into pre-adipocytes during the 12-day experiment and gained two logs between day 0 and day 12 (Figure 4). In mature adipocytes, *M. tuberculosis* and *M. canettii* showed two different kinetics: intracellular *M. tuberculosis* inoculum stabilized between day 3 and 12 p.i. (4 ± 3.66 CFUs gain), whereas *M. canettii* replicated with a 2 × 10^1^ ± 2.5 × 10^1^ CFUs gain between day 3 and 12 p.i. which is significantly higher than *M. tuberculosis* (*p* = 0.01) (Figure 4) but this load remains lower than in pre-adipocytes (Figure 4).

**Figure 5:**
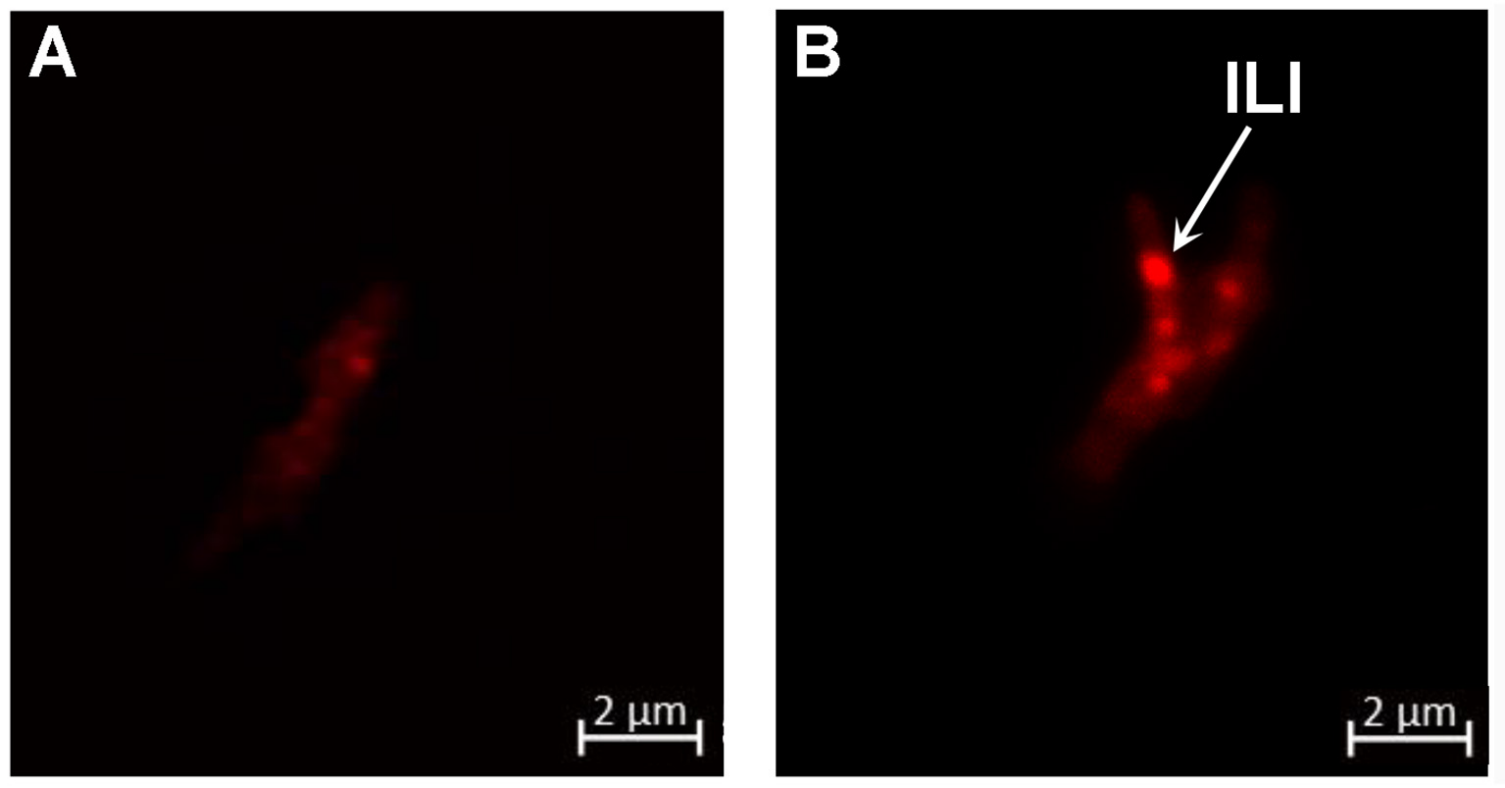
*****M. canettii*** moderately accumulates ILIs inside adipocytes**. Adipocytes infected with *M. canettii* at MOI 5:1 were lysed at day 7 p.i. Intracellular mycobacteria were separated from cellular debris by lysis in water containing 0.1% Triton X-100 and sonication followed by several washes. Nile red staining was performed on **(A)** cultured *M. canettii* used as negative control and **(B)** intra-adipocyte *M. canettii*. Confocal observations at 63x magnification showed a free cytoplasm of cultured *M. canettii* and ILIs seen as bright red spots in the cytoplasm of adipocyte-dwelling *M. canettii* (white arrow). These results are representative of 100 observed mycobacteria in different microscopic fields.

The arrest of *M. tuberculosis*'s replication inside adipocytes has been correlated with a heavy accumulation of ILIs leading to a dormant state (Neyrolles et al., 2006; Kim et al., 2011; Agarwal et al., 2014). To investigate whether this holds true for *M. canettii*, intra-adipocyte bacilli were extracted at day 7 p.i. after Triton X-100 cell lysis and sonication. While Middlebrook 7H10 agar-grown *M. canettii* were free of detectable ILIs, Nile-red staining showed that intracellular *M. canettii* yielded ILIs (Figure 5). Intracellular *M. canettii* presented variable numbers of small ILIs corresponding to 60% ILI^+1^ and 40% ILI^+2^ profiles which were previously described (Caire-Brandli et al., 2014).

#### Intracellular *M. canettii* induce mature adipocyte cytolysis

**Figure 6:**
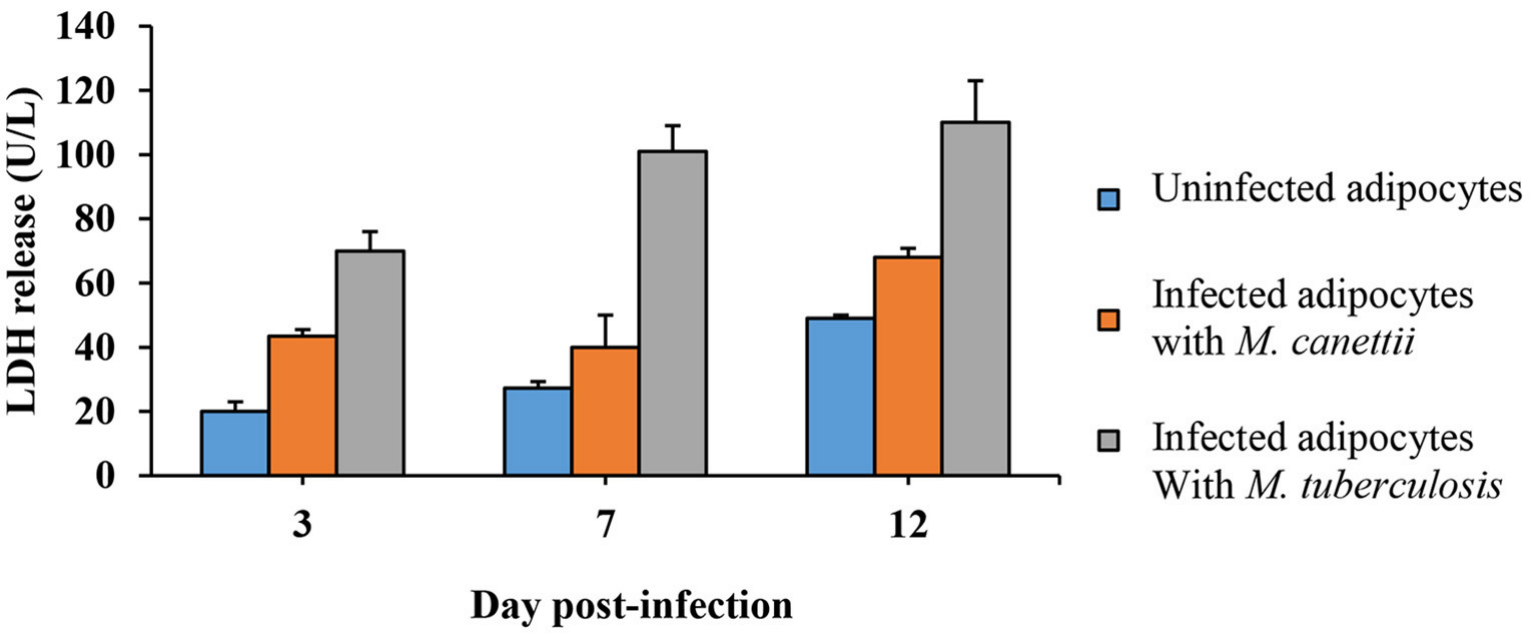
**Quantitative analysis of LDH release**. Culture supernatants of infected adipocytes were collected at day 3, 7, and 12 post-inoculation. LDH concentration was assessed in uninfected adipocytes (blue), *M. canettii* infected cells (orange), and *M. tuberculosis* H37Rv infected cells (gray). Data shown are the mean + standard deviation of three independent measurements.

Microscopic examination indicated that *M. canettii* infection yielded no cytolysis on mature 3T3-L1 adipocytes up to day 7 p.i. but cellular debris were observed at day 12 p.i. while a smaller amount of cellular debris was observed in uninfected control cells. Microscopic observation of *M. tuberculosis*-infected cells clearly showed cellular debris at day 7 p.i. A cytolytic effect of mycobacteria was further investigated by measuring the lactate-dehydrogenase (LDH) in the supernatant of infected and non-infected control adipocytes (see Supplementary Data S1). We observed a progressive increase of the LDH concentration in the three culture supernatants. However, the increase of LDH concentration was significantly higher in *M. canetti*-infected adipocytes than in negative control adipocytes at day 3 p.i. (*p* = 0.002) and day 12 p.i. (*p* <0.001) (Figure 6). In *M. tuberculosis-*infected cells, high rates of LDH concentration measured throughout the 12-day experiment were significantly higher compared to uninfected cells and *M. canettii* infected cells at each time point (Figure 6). Positive and high correlation was noted between the rates of LDH release and the count of CFUs in the case of *M. canettii* and *M. tuberculosis* infection (*r* = 0.90 for *M. canettii* and *r* = 0.88 for *M. tuberculosis*).

## Discussion

Data here reported indicate that *M. canettii* is able to infect, survive and replicate in primary murine white and brown pre-adipocytes which are constantly detected in adipose tissues (Cawthorn et al., 2012) and 3T3-L1 pre-adipocytes and mature adipocytes after *in-vitro* and *ex-vivo* experimental infection. Differentiation of 3T3-L1 cells was induced so as to obtain *in vitro* model of mature white adipocytes (Morrison and McGee, 2015). Data were authenticated by their reproducibility over a triplicate experiment in the presence of negative controls. All experiments with *M. tuberculosis*, here used as a positive control, were in agreement with those previously reported (Neyrolles et al., 2006; Kim et al., 2011; Agarwal et al., 2014). We extended for the first time these observations to brown pre-adipocytes.

Mature adipocytes were infected by *M. canettii* at a lower infectivity than *M. tuberculosis* (Neyrolles et al., 2006; Kim et al., 2011). *M. tuberculosis* binds to adipocytes through scavenger receptors (Neyrolles et al., 2006) but assessing whether this also holds true for *M. canettii* was beyond the scope of our study. Further, *M. tuberculosis* stopped replicating in mature adipocytes in agreement with its previously demonstrated dormant state inside mature adipocytes (Neyrolles et al., 2006; Kim et al., 2011; Agarwal et al., 2014; Rastogi et al., 2016). Unexpectedly, *M. canettii* continuously replicated and accumulated small ILIs. ILIs, previously reported in *M. tuberculosis* (McKinney et al., 2000; Daniel et al., 2004; Deb et al., 2006; Peyron et al., 2008; Russell et al., 2009), *M. bovis* BCG (Low et al., 2009, 2010), *M. leprae* (Mattos et al., 2010, 2011) and *M. smegmatis* (Garton et al., 2002; Dhouib et al., 2011) are here observed for the first time in *M. canettii*. Accordingly, the ILI^+1^/ILI^+2^ and not ILI^+3^ profiles of intracellular *M. canettii* agree with its replicating-state (Caire-Brandli et al., 2014). However, the experimental model used here did not enable us to observe potential long-term dormancy of *M. canettii* in mature adipocytes.

The primary and 3T3-L1 pre-adipocytes also phagocytized *M. canettii* and *M. tuberculosis* as previously shown with other particles (Cousin et al., 1999). More precisely, the amount of intracellular mycobacteria was significantly higher in brown than in white pre-adipocytes, corroborating previous observation of the earlier deposition of *M. bovis* in the BAT after intravenous administration in mice (Mauss and Levy, 1972). *In vivo*, the rich vascular network of BAT (Shimizu et al., 2014) could facilitate the tissue infection by *M. canettii*. Furthermore, the burden of intracellular mycobacteria was higher in pre-adipocytes than in mature adipocytes. Pre-adipocytes are fibroblast-like cells lacking cytoplasmic lipid droplets thus capable of rapidly phagocytosing (Hoffman and Dow, 2016); while mature adipocytes have lost this phagocytic activity (Cousin et al., 1999). Likewise, foamy macrophages loaded with LBs lost the ability to phagocytize mycobacteria compared to undifferentiated non-foamy macrophages (Peyron et al., 2008). These data indicate that pre-adipocytes are preferential targets for MTBC mycobacteria.

In conclusion, our results offer new pieces of information regarding the subtle interplay between MTBC mycobacteria and their hosts: infection of pre-adipocytes and adipocytes is a probable common feature of the MTBC at large, being shared by distantly related *M. tuberculosis* and *M. canettii* (Gutierrez et al., 2005), nevertheless, the intracellular outcome depends on the mycobacteria. While WAT is an unlikely sanctuary for *M. canettii*, the question remains open as for BAT. In the current quest for a yet unknown animal reservoir for *M. canettii*, our observations suggest that any mammal harboring BAT is a candidate reservoir, suggesting new field of investigations.

## Author contributions

SC and MD, conceived and supervised the experiments; Fé.B, performed the experiments; Fa.B, conducted the mice experiment; IP, conducted the electron microscopic observations; PW, was helpful for all confocal observations; SC and MD, and Fé.B analyzed the data and drafted the manuscript. All authors reviewed and approved the manuscript.

## Funding

This study was financially supported by URMITE, IHU Méditerranée Infection, Marseille, France; CNRS and by the A^*^MIDEX project (n°ANR-11-IDEX-0001-02) funded by the “Investissements d'Avenir” French Government program, managed by the French National Research Agency (ANR).

### Conflict of interest statement

The authors declare that the research was conducted in the absence of any commercial or financial relationships that could be construed as a potential conflict of interest.
